# Evidence for *in vitro* and *in vivo* activity of the antimalarial pyronaridine against *Schistosoma*

**DOI:** 10.1371/journal.pntd.0009511

**Published:** 2021-06-24

**Authors:** Erik Koehne, Nina Zander, Miriam Rodi, Jana Held, Wolfgang Hoffmann, Rella Zoleko-Manego, Michael Ramharter, Ghyslain Mombo-Ngoma, Peter G. Kremsner, Andrea Kreidenweiss

**Affiliations:** 1 Institute of Tropical Medicine, University Hospital Tübingen, Tübingen, Germany; 2 Centre de Recherches Médicales de Lambaréné, Lambaréné, Gabon; 3 Department of Tropical Medicine, Bernhard Nocht Institute for Tropical Medicine & I. Dep. of of Medicine, University Medical Center Hamburg-Eppendorf, Hamburg, Germany; 4 German Center for Infection Research (DZIF), partner site Tübingen, Tübingen, Germany; Queen’s University Belfast, UNITED KINGDOM

## Abstract

**Background:**

Schistosomiasis is highly prevalent in Africa. Praziquantel is effective against adult schistosomes but leaves prepatent stages unaffected—which is a limit to patient management and elimination. Given the large-scale use of praziquantel, development of drug resistance by Schistosoma is feared. Antimalarials are promising drugs for alternative treatment strategies of Schistosoma infections. Development of drugs with activity against both malaria and schistosomiasis is particularly appealing as schistosome infections often occur concomitantly with malaria parasites in sub-Saharan Africa. Therefore, antiplasmodial compounds were progressively tested against Schistosoma *in vitro*, in mice, and in a clinical study.

**Results:**

Amongst 16 drugs and 1 control tested, pyronaridine, methylene blue and 5 other antimalarials were highly active *in vitro* against larval stage schistosomula with a 50% inhibitory concentration below 10 μM. Both drugs were lethal to *ex vivo* adult worms tested at 30 μM with methylene blue also active at 10 μM. Pyronaridine treatment of mice infected with *S*. *mansoni* at the prepatent stage reduced worm burden by 82% and cured 7 out of 12 animals, however in mice adult stages remained viable. In contrast, methylene blue inhibited adult worms by 60% but cure was not achieved. In an observational pilot trial in Gabon in children, the antimalarial drug combination pyronaridine-artesunate (Pyramax) reduced *S*. *haematobium* egg excretion from 10/10 ml urine to 0/10 ml urine, and 3 out of 4 children were cured.

**Conclusion:**

Pyronaridine and methylene blue warrant further investigation as candidates for schistosomiasis treatment. Both compounds are approved for human use and evidence for their potential as antischistosomal compounds can be obtained directly from clinical testing. Particularly, pyronaridine-artesunate, already available as an antimalarial drug, calls for further clinical evaluation.

**Trial registration:**

ClinicalTrials.gov Identifier NCT03201770.

## Author summary

Praziquantel is still the only drug in use for the treatment of all *Schistosoma spp*. and is exclusively active against the adult life cycle stage, since schistosomes in the prepatent period of up to eight weeks are not affected by the drug. Although resistance to praziquantel has not been confirmed and its existence remains controversial, some countries have identified clinical schistosome isolates with reduced sensitivity to praziquantel, after deployment in mass drug administration programs. The need for a new antischistosomal compound is urgent, ideally exhibiting broad activity against all stages of the parasite’s life cycle present in humans. After testing a series of antiplasmodial compounds, the authors found that several compounds also exhibited antischistosomal activity at various life cycle stages of the worms, including pyronaridine and methylene blue, both compounds already approved for human use. A pilot trial with pyronaridine-artesunate done in Gabon showed the first promising results against Schistosoma infections.

## Introduction

Schistosomiasis is an infectious disease caused by parasitic flatworms of the genus *Schistosoma*. WHO estimated that approximately 100 million people were treated for schistosomiasis in 2018, with about 90% of cases occurring in Africa, and approximately 290 million people receiving preventive treatment [1]. Praziquantel (PZQ), the only drug for the treatment of all *Schistosoma spp*., including *Schistosoma mansoni*, *S*. *haematobium*, and *S*. *japonicum*, is exclusively active against the adult life cycle stage. Schistosomes in the prepatent period of up to eight weeks are not affected which is a limit to schistosomiasis control and elimination efforts by PZQ mass administration [2,3]. Resistance to PZQ has not been confirmed and its existence remains controversial, yet, clinical schistosome isolates with reduced sensitivity have been identified in Egypt and Senegal after PZQ deployment in mass drug administration programs [4–6]. The need for a new antischistosomal compound is urgent, optimally exhibiting broad activity against all stages of the parasite’s life cycle present in humans.

Drug repurposing is a rapid and efficient strategy to identify compounds with new therapeutic targets [7]. Interestingly, schistosomes and malaria parasites both degrade blood/hemoglobin and depend on intracellular mechanisms to protect themselves from heme toxicity; a molecular pathway inhibited by several antimalarial compounds [8,9]. In Africa, individuals are often co-infected with *Schistosoma spp*. and *Plasmodium spp*. since both parasites have a large geographical overlap in endemicity. Antiplasmodial compounds approved for use in humans may serve as “low hanging fruits” for the development of new antischistosomal interventions either as antimalarial treatments with an add-on effect against schistosomes or as a starting point for a schistosomiasis drug development program.

Indeed, antimalarials such as artesunate, artemether, and mefloquine have been tested, as a monotherapy or in combination, in clinical trials for their antischistosomal potency [10–12]. Of particular interest is their activity against young schistosomes, as identified in preclinical studies [13,14]. Differences in the design and quality of the studies carried out make it difficult to draw an overall conclusion on the antischistosomal efficacy of artemisinins and mefloquine [15,16]. However, given together with PZQ, all life cycle stages in humans could be targeted [17,18]. Artemisinin and related derivates (i.e. artesunate, artemether) constitute the standard of care for malaria treatment that is an artemisinin-based combination therapy (ACT). An ACT is formed by an artemisinin derivate combined with a long-acting partner drug of a different drug class. Amongst the six ACTs on the market, pyronaridine-artesunate was registered recently with a granule’s formula also available for treatment of small children. Indication towards an add-on antischistosomal effect of an ACT drug component could be an interesting starting point for further clinical evaluation. Several antimalarials have also been tested preclinically for activity against *Schistosoma* within individual small scale studies and/or library screenings projects [19,20]. Here, we tested a series of antiplasmodial compounds head-to-head in a hierarchical workflow for their antischistosomal activity and chose the most promising compounds for *in vivo* testing [21].

## Results

In total, 16 compounds with reported antiplasmodial activity were tested for activity against *S*. *mansoni*. The drugs were tested in a hierarchical workflow to downselect candidates with antischistosomal activity starting with *in vitro* drug sensitivity assays on human larval stage schistosomes, schistosomula, (step 1) followed by testing of *ex vivo* adult worms (step 2), and promising compounds were assayed in *S*. *mansoni* infected mice (step 3). An important aspect was to identify molecules with activity against juvenile worms. This stepwise approach helps to reduce animal testing. In addition, preliminary data of an observational study assessing the activity of pyronaridine-artesunate against urinary schistosomiasis in children in Gabon were analyzed (step 4).

### *In vitro* schistosomula assays (step 1)

**Fig 1:**
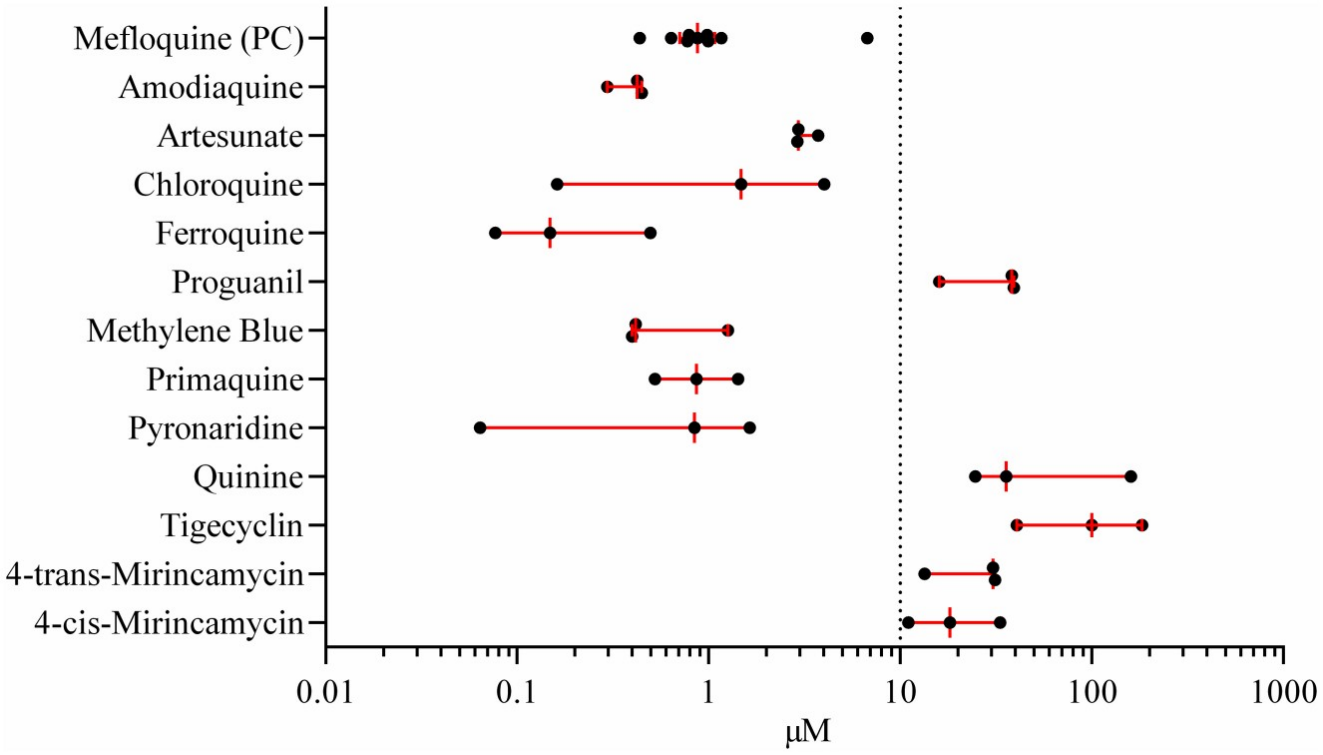
*In vitro* schistosomula assays (step 1). Dot plot of individual IC50 values of 12 drugs and mefloquine control is shown. Each compound was tested in 3 independent, *in vitro* schistosomula experiments (except for MQ, N = 9) and individual IC50s (filled circles) are shown. Viability of schistosomula after 7 days drug exposure was assessed by microscopy. For four drugs (atovaquone, cycloguanil, doxycycline, clindamycin) the IC50 could not be calculated as no inhibition was observed. The IC50 is given in μM. Bar and lines indicate median IC50 and IQR. Dotted line: Threshold for drug activity is an IC50 < 10 μM. IQR: Interquartile range.

Most of the compounds were registered antimalarial drugs of diverse pharmacological classes including antibiotics (S1 and S2 Tables) and were tested head-to-head within the same assay set-up. In the analysis only experiments passing the following quality criteria were included: schistosomula viability > 80% after 7 days and the 50% inhibitory concentration (IC50) of mefloquine below 2 μM (S1 Fig). Of the 16 compounds tested, 7 had an IC50 below 10 μM (Fig 1). None of the antibiotics were active (Table 1). The positive control drug mefloquine was confirmed to be highly active and, interestingly, together with methylene blue, were the only 2 drugs with a rapid onset of activity (IC50 below 10 μM after 72 h).

**Table 1:** Assay-dependent IC50s of compounds against schistosomula (step 1).

Drug	Microscopy at 72 h	Microscopy day 7	Resazurin day 7	Lactate day 7
Mefloquine	4.9 (2.7–7.8)	0.8 (0.7–1.1)	0.7 (0.6–1.9)	1.7 (0.7–4.1)
Amodiaquine	no inhibition	0.4 (0.3–0.4)	0.2 (0.1–0.3)	no inhibition
Artesunate	28.4 (14.3–30.1)	2.9 (2.9–3.7)	1.7 (1.7–3.4)	12.5 (7.9–16.6)
Atovaquone	no inhibition	no inhibition	no inhibition	no inhibition
Chloroquine	no inhibition	1.5 (0.2–4.0)	2.7 (0.4–4.2)	no inhibition
Ferroquine	no inhibition	0.1 (0.1–0.5)	0.6 (0.5–0.8)	no inhibition
Cycloguanil	no inhibition	no inhibition	no inhibition	no inhibition
Proguanil	71.6 (41.3–99.6)	38.2 (16.0–39.3)	15.7 (19.0–13.6)	45.9 (33.3–58.4)
Methylene blue	1.4 (1.9–3.7)	0.4 (0.4–1.3)	#	0.3 (0.3–0.4)
Primaquine	12.7 (5.2–45.3)	0.9 (0.5–1.4)	0.5 (0.2–1.0)	6.1 (5.0–7.2)
Pyronaridine	71.6 (41.3–99.6)	0.8 (0.1–1.6)	0.5*	1.0*
Quinine	no inhibition	35.9 (24.7–160.2)	26.3 (21.0–70.0)	no inhibition
Doxycycline	no inhibition	no inhibition	no inhibition	no inhibition
Clindamycin	no inhibition	no inhibition	no inhibition	no inhibition
cis-Mirincamycin	33.2 (22.4–45.3)	18.2 (11.0–33.2)	13.4 (12.4–29.3)	18.7 (10.0–41.3)
trans-Mirincamycin	33.2 (31.2–67.2)	30.6 (13.4–31.2)	19.3 (17.9–28.5)	33.6 (17.3–35.7)
Tigecycline	125.0 (59.5–125.0)	100.0 (40.1–83.0)	no inhibition	no inhibition

*In vitro* viability of *S*. *mansoni* schistosomula was assessed by microscopy after 72 h and at day 7 after drug exposure, respectively, and by resazurin assay and lactate assay after 7 days only. Median (IQR) in μM is reported. Mefloquine served as positive control.

^#^ could not be measured due to drug colour interference with the reader.

* IC50 value from one assay. Each drug was independently tested at minimum three times.

There is no *gold standard* methodology to determine the worms´ viability after drug exposure to report a compound´s antischistosomal activity. Microscopy based classification is dependent on the reader´s subjective judgement. To minimize observer bias, every assay was not only analyzed by microscopy, but after 7 days drug exposure outcomes were concomitantly analyzed by resazurin and lactate assays. Resazurin measurement [22] reflects aerobic respiration and lactate detection is a surrogate marker for glucose metabolism, [23] both indicating cell viability. Overall, IC50s obtained by microscopy and by resazurin assay on day 7 were in agreement for active compounds with lower IC50s (Table 1 and S2 Fig). Pyronaridine was active, but only one experiment resulted in a sigmoidal dose-response curve allowing for the calculation of the IC50 at the concentration range tested (S5 Fig). Interestingly, the lactate assay identified compounds with a rather rapid onset of inhibition(IC50 ≤ 10 μM after 72 h drug exposure) as found by microscopy at 72 h (mefloquine, methylene blue, primaquine (IC50 of 12.7 μM). Drugs which exerted an IC50 below 10 μM after 7 days only, (amodiaquine, chloroquine) were not found active by lactate assay.

### *Ex vivo* adult worm assays (step 2)

Methylene blue and pyronaridine were further tested for their *in vitro* activity against adult *S*. *mansoni*. Methylene blue at 10 μM for 7 days was highly active, whereas pyronaridine was active at 30 μM (Table 2). Both drugs rapidly killed all worms within 24 h at 30 μM. Irreversibility of drug effects was controlled by continued culture for 7 days after drug/medium replacement by fresh medium only (S3 Fig and S1 Videos).

**Table 2:** Drug activity against *ex vivo* adult worms (step 2).

Drug	Concentration in μM	No. of worms tested	% affected	% dead
No drug	NA	12	0	0
DMSO	0.1%	10	0	0
Praziquantel	1	12	0	100
	5	17	35	59
Methylene blue	10	12	0	100
	30	12	0	100
	30*	6	0	100
	5	15	0	60
Pyronaridine	10	11	45	55
	30	12	0	100
	30*	6	0	100

Methylene blue, pyronaridine, and praziquantel (positive control) were exposed to the respective concentrations for 7 days (* for 24 h) followed by a 7 days drug wash-out *in vitro* culture.”No drug” and DMSO were the negative controls. Pooled data obtained from 3 experiments per drug are displayed.

### *In vivo* drug activity in *S*. *mansoni*-infected mice (step 3)

**Fig 2:**
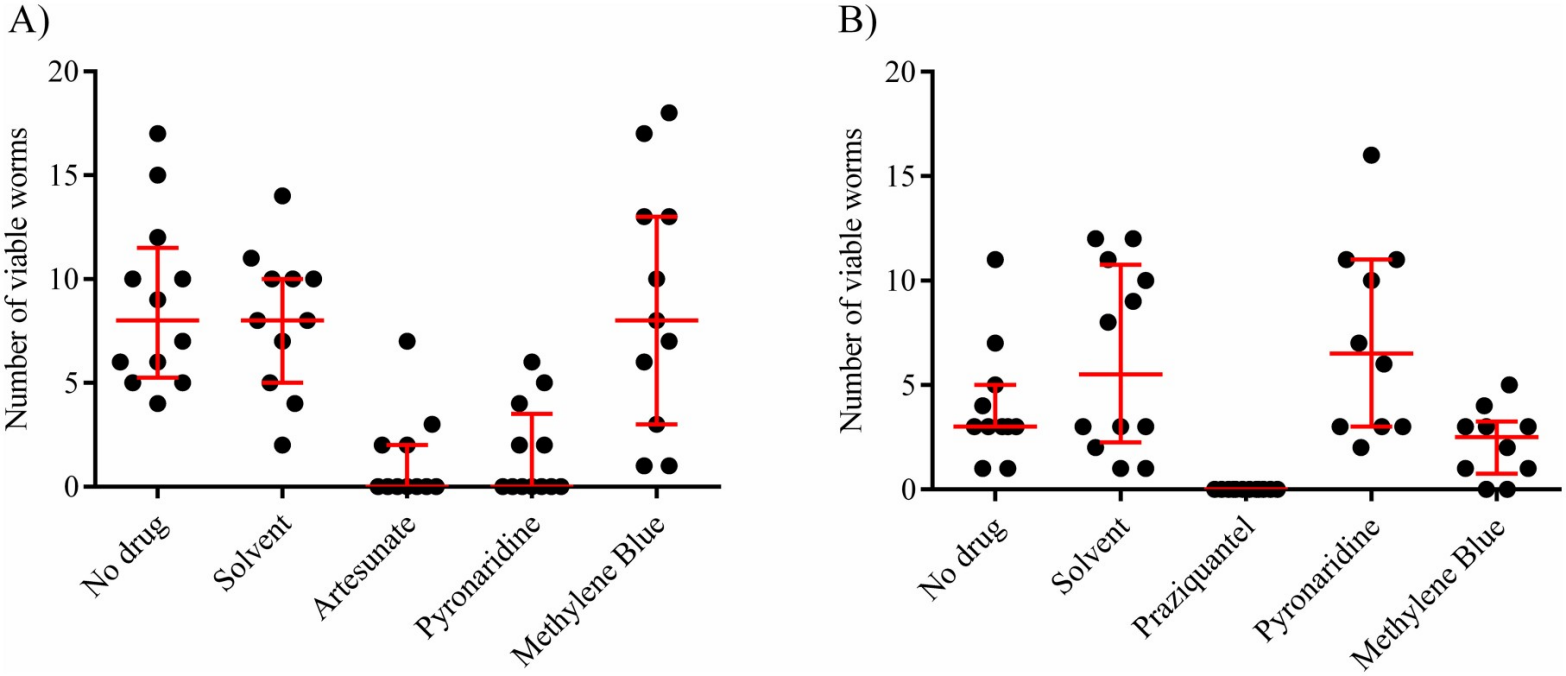
Drug activity in *S*. *mansoni*-infected mice (step 3). Mice were treated with respective compounds after A) 14 days (juvenile worms) and B) 9 weeks (adult worms) of *S*. *mansoni* infection, respectively. Mice were killed and adult worms were recovered and counted. MB: methylene blue, PY: pyronaridine, AS: artesunate, PZQ: praziquantel. Each data point (filled circle) represents one mouse. Thick bar: median, whiskers: IQR. A statistically significant difference (P < 0.05) to no drug control was detected for A) AS and PY, and for B) PZQ, respectively. Results from two independent experiments are shown for A and from one experiment for B.

Pyronaridine and methylene blue were chosen for further evaluation based on their *in vitro* activities against schistosomula, adult worms, and review of the literature (S2 Table) *In vivo* potency of compounds was tested against mice infected with *S*. *mansoni* at the juvenile stage (14 days after mice were infected with *S*. *mansoni*) as well as against the adult stage (9 weeks after *S*. *mansoni* infection). Artesunate and praziquantel were included as positive control drugs with known activity against juvenile schistosomes and adult worms, respectively [24,25]. Pyronaridine (500 mg/kg/day given for 5 days) was highly active against juvenile stages with a cure rate of 58%, whereas methylene blue (100 mg/kg/day given for 5 days) did not show an effect. For pyronaridine, the number of worms was substantially reduced (82%) reaching a comparable efficacy level as artesunate (79%) (Fig 2A and Table 3). No schistosome eggs (liver granulation) were observed in the livers of pyronaridine treated mice in contrast to methylene blue treated mice which displayed eggs in numbers as high as in the control mice (S4 Fig). Although cure was not achieved, methylene blue reduced the adult worm burden considerably (57%), which was not observed for pyronaridine (Fig 2B and Tables 4 and S4).

**Table 3:** Activity against juvenile *S*. *mansoni* in mice.

Juvenile worms (2 weeks old)								
Drug	Dose in mg/kg	No. of mice	No. of worms/mouse recovered Median (IQR)			Cure in %	WBR in %	LGS Median (IQR)
			Total	Males	Females			
No drug	NA	12	8.0	5.5	2.0	NA	0	2.5
			(5.3–11.5)	(4.0–9.0)	(1.0–4.5)			(2.0–3.0)
Solvent	NA	12	9.0	8.0	1.5	NA	0	3.0
			(5.5–10.8)	(4.0–9.8)	(0–2.8)			(2.3–3.0)
Artesunate	300	12	0.0	0.0	0.0	58	79	0.0
			(0.0–2.8)	(0.0–2.8)	(0–0.8)			(0.0–0.8)
Pyronaridine	500	12	0.0	0.0	0.0	58	82	0.0
			(0.0–3.5)	(0–1.8)	(0–1.0)			(0.0–0.8)
Methylene blue	50	12*	8.0	7.0	2.0	0	1	3.0
			(3.0–13.0)	(2.0–10.0)	(0.0–4.0)			(3.0–3.0)

Mice infected with *S*. *mansoni* for 2 weeks (worms are in the juvenile stage) were treated with methylene blue (MB) or pyronaridine (PY). Negative control treatment: No drug and solvent, positive control treatment: Artesunate (AS). WBR is referred to the “No drug” group. WBR: Worm burden reduction, LGS: Liver granulation score.

*1 mouse died during the treatment week. Compounds were tested in two independent experiments.

**Table 4:** Activity against adult *S*. *mansoni* in mice.

Adult worms (9 weeks old)								
Drug	Dose in mg/kg	No. of mice	No. of worms/mouse recovered Median (IQR^a^)			Cure in (%)	WBR in %	LGS Median (IQR^a^)
			Total	Males	Females			
No drug	NA	6	6.0	4.0	2.0	NA	0	3.0
			(2.5–15.0)	(1.8–8.8)	(0.8–6.3)			(3.0–3.0)
Solvent	NA	6	10.5	5.5	5.0	NA	0	3.0
			(6.3–12.0)	(4.0–6.3)	(2.3–5.3)			(2.8–3.0)
Praziquantel	500	6	0.0	0.0	0.0	100	100	3.0
			(0.0)	(0.0)	(0.0)			(2.8–3.0)
Pyronaridine	500	6*	11.0	6.0	4.0	0	0	3.0
			(8.5–13.5)	(5.5–7.0)	(3.0–7.0)			(3.0–3.0)
Methylene blue	50	6*	3.5	2.5	1.0	0	57	3.0
			(3.0–4.8)	(2.0–4.5)	(0.3–1.0)			(2.3–3.0)

Mice infected with *S*. *mansoni* for 9 weeks (worms are in the adult stage) were treated with methylene blue (MB) or pyronaridine (PY). Negative control treatment: No drug and solvent, positive control treatment: Praziquantel (PZQ). WBR is referred to the “No drug” group. WBR: Worm burden reduction, LGS: Liver granulation score.

* 1 mouse died during the treatment week.

^a^ Interquartile range: IQR. Results from one experiment are shown (a second experiment did not produce comparable infection rates of mice and results were excluded here but can be found in S4 Table).

### Observational pilot study in Gabonese patients (step 4)

**Fig 3:**
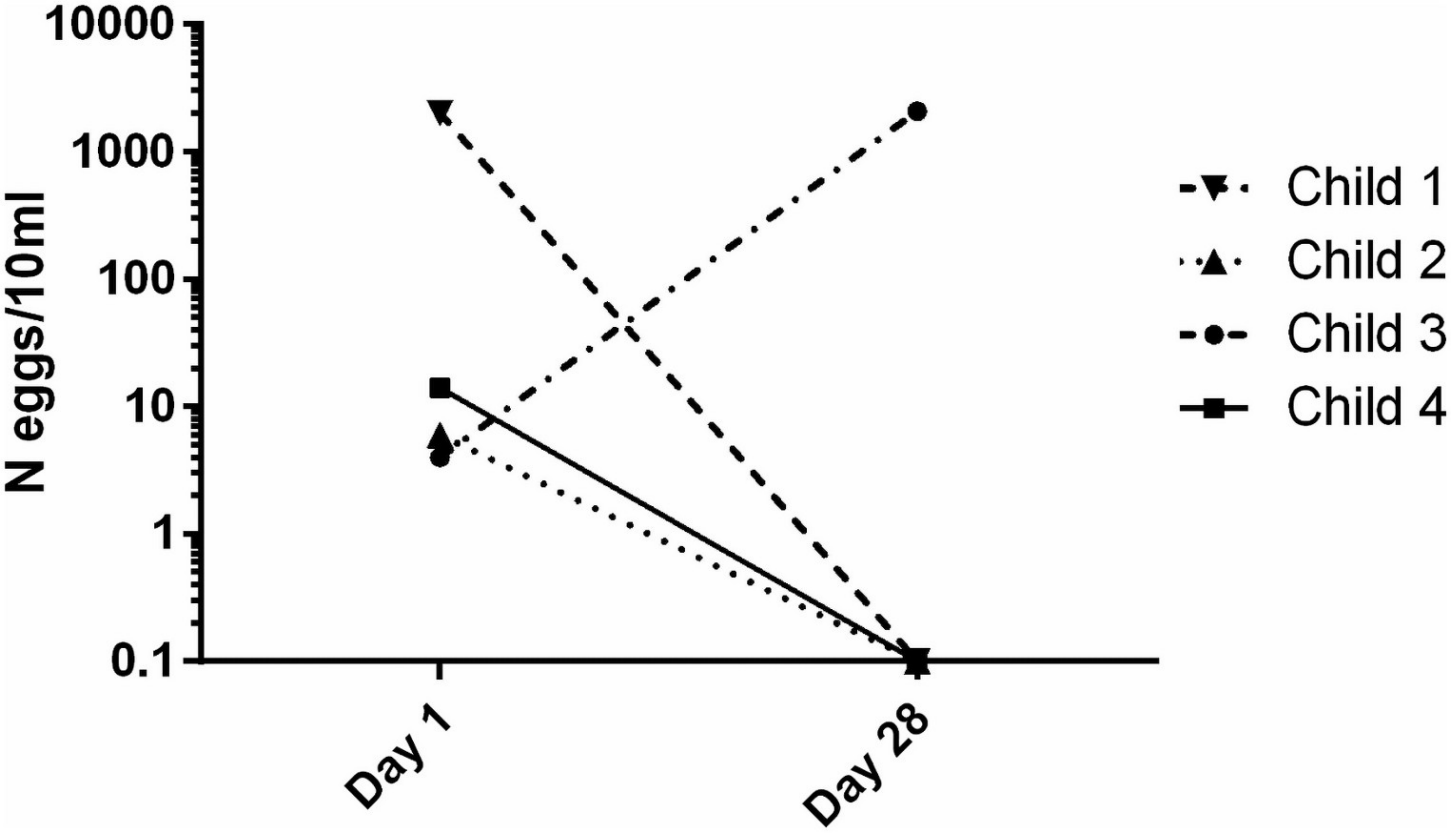
Observational study in Gabonese patients (step 4). Individual treatment response. Children excreting *S*. *haematobium* eggs were treated with pyronaridine-artesunate starting at day 1. After 28 days, urines were analyzed again and number of eggs/10 ml urine was reported.

Ultimately, data from *S*. *haematobium* infected children who received pyronaridine-artesunate within a non-randomized, observational, study for uncomplicated malaria were analyzed for efficacy of pyronaridine-artesunate against urinary schistosomiasis. This preliminary proof-of-concept exploratory analysis was done for 6 children at the trial site CERMEL/Gabon. Egg excretion was assessed 28 days after treatment start in 4 children (2 children were lost to follow-up) (S3 Table). Median number of excreted eggs was 10/10 ml of urine (IQR: 5–1528) at the day of treatment start and 0/10 ml (IQR: 0–1570) 28 days later. Cure was achieved in 3 out of 4 children (Fig 3). Microhematuria and proteinuria were resolved in 50% and excretion of leucocytes was resolved in 75% of the children.

## Discussion

Schistosomiasis treatment, prevention, and control suffers from a lack of approved treatment options besides praziquantel as the standard of care. As concomitant schistosome and malaria parasite infections are frequent in Africa, a comprehensive assessment of antimalarial drugs for their potency against schistosomiasis could be an efficient starting point for the development of new intervention strategies [26].

We tested a series of compounds with known antiplasmodial activity for their inhibitory effect against *S*. *mansoni*. Out of the 16 compounds investigated plus mefloquine and artesunate used here as controls, 9 drugs (including mefloquine, artesunate, amodiaquine, and atovaquone) have been previously screened for their activity against various life cycle forms of *S*. *mansoni*. To consolidate evidence of a molecule´s potential for further antischistosomal drug development, we did a head-to-head *in vitro* evaluation of antimalarials against larval, prepatent schistosomes, and a selection of candidates were further tested *in vitro* against *ex vivo* adult worms and *in vivo* in mice. The first stage-gate was set to an IC50 of 10 μM against schistosomula *in vitro* to [21] downselect the number of candidates and to reduce animal involvement. Our approach confirmed the reported antischistosomal activity of mefloquine [27], artesunate, and primaquine [28] when schistosomula stages were exposed for 7 days and found chloroquine and ferroquine highly active. We confirmed the insensitivity of schistosomes to antibiotics although some interesting antischistosomal effects were observed for both isomers of mirincamycin.

Amodiaquine and methylene blue, the latter so far only used for staining and visualization of the worms [29,30] were active against *in vitro* schistosomula. We also confirmed activity of pyronaridine against schistosomula and found both, pyronaridine and methylene blue, rapidly inhibitin*g ex vivo* adult worms tested *in vitro*. Despite similar assay conditions (24 h approx. 30 μM drug exposure), Panic et al. [19] found methylene blue and pyronaridine not efficacious against *ex vivo* adult worms although these results are not further detailed, but consequently refrained from mouse testing. This discrepancy in assay outcomes underlines the need of standardized and validated assay methodology and repetitive antischistosomal testing of molecules by various groups. This also includes harmonization of culture media as serum binding properties of compounds may impact drug activity [31].

Methylene blue, a registered drug to treat methemoglobinemia caused by drug or toxin exposure and in development as an antimalarial [32,33], and pyronaridine, approved as a malaria treatment in a fixed combination with artesunate (Pyramax) were further tested in mice infected with *S*. *mansoni* at the juvenile or adult stage, respectively, at the time of treatment administration. We found pyronaridine to be active against the juvenile forms with little to no activity against adult worms, which is in line with Keiser et al. [13]. No eggs were found in the livers of dissected mice treated with pyronaridine indicating a high potency against young life cycle forms. Methylene blue, on the other hand, was very potent against the adult worms *in vivo*. Methylene blue has been found to act by preventing the polymerization of heme into hemozoin in *Plasmodium* parasites [34] and studies on the mode of action of pyronaridine report inhibition of β-hematin production and glutathione-dependent heme degradation [35,36]. This makes both compounds interesting candidates for further development of novel schistosomiasis treatments since they have the potential to inhibit vital heme-related processes of *P*. *falciparum* and potentially also of *S*. *mansoni*.

Schistosomiasis drug discovery programs are still hampered by the lack of robust, scalable, and quantitative read-outs of schistosome viability after drug exposure that limits phenotypic whole organism screens. We recently identified the energy metabolism of schistosomes as a new target to quantify the efficacy of a test compound [23] which may prove to be especially helpful for schistosomula screens. Viable schistosomes rely on glycolysis of glucose to generate energy and excrete lactate that accumulates in the surrounding medium. Interestingly, drugs with a rapid onset of activity (exerting 72 h IC50 by microscopy below/around 10 μM) were also identified active by lactate assay and compounds with lower activities were confirmed by lactate assay outcomes (either high IC50 values or no IC50 could be calculated). Detection of lactate levels might reflect how rapidly the worm´s glycolysis is inhibited and could indicate early on set of drug activity. Resazurin assays report worm viability/death at the end of drug exposure and the assay reliably identified active compounds when compared against microscopy after 7 days drug exposure. Another hurdle to drug development is the limited translation of efficacy data derived from animal models into clinical efficacy. PZQ, the only schistosomiasis drug, requires an oral dose of 400 mg/kg in *S*. *mansoni* infected mice [13], whereas only 40 mg/kg is administered to humans. Methylene blue and pyronaridine were tested active at 100 mg/kg and 500 mg/kg respectively, in *S*. *mansoni* mice. Although for malaria treatment both drugs are used at considerably lower doses (approximately 20 mg/kg for 3 days [37] for methylene blue and 1 tablet of 180 mg pyronaridine (plus 60 mg artesunate) once daily for 3 days for children with a weight of 20 to max. 24 kg), their effectiveness against schistosomes requires testing in clinical trials.

Pyronaridine is the long acting partner drug of artesunate in the artemisinin-based combination therapy (ACT) that was recently recommended by WHO for the treatment of uncomplicated malaria [38]. Pyronaridine has an estimated terminal elimination half-life of around 14 days [39] in contrast to artesunate with approximately 1 h [40]. Clinical trials testing mefloquine [41] and ACTs like artesunate-amodiaquine [42], artesunate-sulfalene–pyrimethamine, or artemether-lumefantrine [43,44] in patients concomitantly infected with *P*. *falciparum* and *Schistosoma spp*. reported a beneficial secondary antischistosomal efficacy, e.g. reaching an egg reduction rate of 98% for mefloquine. However, treatment trials specifically designed to determine ACT efficacy in schistosomiasis found less promising results [26]. In our first explorative analysis of data obtained from a non-randomized, observational antimalarial treatment trial done in Gabon, 3 out of 4 children who received pyronaridine-artesunate for treatment of uncomplicated malaria were cured from *S*. *haematobium* infections. We are aware that the infection intensity of the children was low making them more likely to be cured from a *Schistosoma* infection. Prospectively conducted randomized, controlled, blinded trials are needed to properly characterize the effect of pyronaridine-artesunate against (concomitant) *Schistosoma* infections. An important aspect is the investigation of drug activity against the juvenile worms, which requires a specifically designed experimental model. Such clinical work can relatively easily be done with compounds approved for use in humans and is a straightforward approach to conclude on the antischistosomal potential of pyronaridine (at least at the antimalarial dosage). From our work, we have evidence towards activity against young worms, but it is not yet clear if there is also a beneficial effect against adult worms. Despite a detrimental effect of pyronaridine on *in vitro* cultured *ex vivo* adult worms (assay read-out after 14 days of culture where 7 days drug exposure was followed by a 7 days drug washout phase), no inhibition of adult worms were observed in Schistosoma infected mice. Methylene blue is another candidate which warrants future clinical investigation of a concomitant beneficial effect on schistosomes but more preclinical data would be helpful for decision making.

Our results demonstrate promising antischistosomal properties of a selection of antimalarials, especially pyronaridine, which was highly active against juvenile *S*. *mansoni* worms, and methylene blue, which was active against adult *S*. *mansoni* worms. First hints from clinical data towards pyronaridine-artesunate activity against urinary schistosomiasis are reported. These results demonstrate the potential of antiplasmodial compounds, especially pyronaridine and methylene blue, as candidate compounds for the development of novel schistosomiasis therapies, or at least as lead compounds for even more active derivatives.

## Methods

### Ethics statement

Animal experiments were conducted in accordance with German laws after approval by the local authorities (Regierungspräsidium Tübingen, Germany, animal testing license T1/16). The clinical study was approved by the institutional ethics committee of CERMEL (reference number: CEI- 006/20189) and was registered at ClinicalTrials.gov Identifier NCT03201770. The trial was conducted according to ICH-GCP and national guidelines. Written informed consent was obtained from the parents of the children.

### *Schistosoma* life cycle

The life cycle of *S*. *mansoni* (Puerto Rico PR-1 strain) is routinely maintained in NMRI mice (female, 20–22 g) and in *Biomphalaria glabrata* snails. Mice were infected percutaneously by bathing the mice´ feet for 1 hour in cercaria containing waters (50 cercaria per mouse to keep the lifecycle or 100 cercaria for *in vivo* studies). To obtain schistosomula for *in vitro* drug testing, cercaria shed from infected snails were manually transformed. Adult worms were obtained from mice after 8–10 weeks of infection. All procedures were previously described [23].

### Compounds

Artesunate, mefloquine hydrochloride, amodiaquine dihydrochloride dihydrate, chloroquine diphosphate, primaquine phosphate, quinine hemisulfate, atovaquone, methylene blue, pyronaridine tetraphosphate, doxycycline hyclate, clindamycin and praziquantel were purchased from Sigma-Aldrich. Ferroquine was obtained from Sanofi-Synthelabo, proguanil and cycloguanil from Jacobus Pharmaceutical Company, tigecycline from Wyeth, and mirincamycin hydrochloride enantiomers from Maldevco [45]. All compounds were dissolved in sterile DMSO except for quinine for which methanol was used and pure M199 medium (without additives) was used to dissolve proguanil, cycloguanil, clindamycin, and pyronaridine. The stock concentration was 50 mM for artesunate, amodiaquine, chloroquine, atovaquone, quinine, and primaquine, and 100 mM for praziquantel, proguanil, cycloguanil, methylene blue, pyronaridine, clindamycin, doxycycline, and mirincamycin enantiomers, respectively. Mefloquine was dissolved to 24 mM and ferroquine to 12.5 mM. All stocks were freshly prepared for the study and stored at -20°C. Maximum concentration of the solvent (DMSO, methanol) in the *in vitro* assays did not exceed 0.8% and did not interfere with parasite viability.

### *In vitro* drug sensitivity testing of schistosomula (step 1)

Compounds were pre-dosed (25 μl/well) in 96-well flat-bottom plates in 2-fold or 3-fold serial dilutions including wells with schistosomula culture medium only (SCM: phenol-red free medium 199 (Thermo Fisher Scientific), 1% decomplemented fetal bovine serum (FBS, Gibco), and 200 μg/mL streptomycin/200 U/l penicillin (Gibco)). The range of drug concentrations tested is given in S1 Table. Mature schistosomula (24 h old) in SCM were added to pre-dosed wells to obtain 100 schistosomula per well in a total volume of 225 μl/well. The exact number of schistosomula per well was counted by microscopy. Schistosomula drug assays were kept at 37°C and 5% CO_2_.

Viability of schistosomula per well was assessed by three different methodologies: i) assessment of morphology and motility by microscopy after 3 and 7 days of drug exposure, ii) resazurin assay, and iii) lactate assays, both done after 7 days of assay start. For the microscopic read-out, all schistosomula per well were visually judged using an inverted microscope (Nikon eclipse Ti 20x objective) for motility and morphology and classified either as viable (movement and normal appearance) or dead (severe morphological changes, e.g., granularity, blebbing, and/or no movement within 10 s of observing an individual worm)). Viability was expressed as % of total worms/well. The resazurin assay was done by removing 20 μl of supernatant/well and adding 20 μl AlamarBlue reagent (ThermoFisher Scientific cat. no. DAL1025). The plate was incubated at 37°C, 5% CO_2_ and read in a fluorometer at 530 nm/590 nm after 24 h. Of the removed supernatant, 10 μl were further analyzed to quantify lactate levels as described earlier [23]. Briefly, fluorometric L-lactate assay kit (Abcam cat. no. ab65330) and 96-well, black-sided, optical clear-bottom plates (Corning cat. no. 3340) were used to quantify lactate levels following manufacturer´s specifications with minor modifications. After 40 min of incubation at room temperature, the plate was read by the fluorometer at 530 nm/590 nm. Both assays included SCM to determine the background resazurin and lactate levels, respectively, for normalization of readings of the experiment measurements.

### Drug sensitivity testing of *ex vivo* adult worms (step 2)

For the *in vitro* drug sensitivity assays, *ex vivo* adult worms were set in 24 well-plates with 2 worms (1 pair or 1 separated pair, or 2 individual worms)/well in 1 ml adult worm culture medium (ACM; phenol-red free RPMI, 100 U/ml penicillin plus 100 μg/ml streptomycin, 5% FBS) and were kept at 37°C and 5% CO_2_. Worms Parasites were adapted to culture conditions for 24 h. Every experiment included 1 μM PZQ as positive control and 0.1% DMSO and ACM only as negative controls. Drugs were tested at concentrations of 1 μM, 5 μM, 10 μM, and 30 μM for 7 days, and 30 μM for 1 day. To control for irreversibility of drug effects, drug/medium mixture was replaced by fresh ACM (without drug) and *in vitro* culture was continued for an additional 7 days (drug wash-out). Viability of the worms was assessed by microscopy at the end of the drug wash-out period. Viability was categorized into 3 stages based on the worms´ motility (normal movement = viable, some/little movement = affected, no movement = dead) assessed via an inverted microscope (Wild Heerbrugg M7A Stereo Zoom microscope 6x-31x).

### *In vivo* mouse studies (step 3)

Mice infected with *S*. *mansoni* at the juvenile life cycle stage (2 weeks after infection, 60 mice in groups of 6 animals) or at the adult stage (9 weeks after infection, 30 mice in groups of 6 animals) were treated with the study drugs dissolved in 7% Tween 80 and 3% ethanol in PBS and administered via oral gavage. Methylene blue (twice per day 50 mg/kg) and pyronaridine (500 mg/kg) were given for 5 consecutive days. Positive control drugs were artesunate (5 days 300 mg/kg/day) or praziquantel (once 300 mg/kg) for the juvenile and the adult life cycle stages, respectively and negative control was solvent (10 ml/kg). After 8 weeks or 2 weeks following treatment start, mice were sacrificed and recovered worms were counted to determine efficacy against juvenile or adult stages, respectively. Liver granulation was determined by counting the number of *S*. *mansoni* eggs on the outer surface of the liver and classified into no granulation or 0 eggs (score of 0), low granulation or 1–10 eggs (score of 1), medium granulation or 11–99 eggs (score of 2), and high granulation or ≥100 eggs (score of 3). Two independently conducted experiments were done for the juvenile stage as well as for the adult worms (for adult worms results from one experiment are shown, as infection rates of mice were not sufficient in the repeated experiment (shown in S4 Table).

The sample size (number of mice/group) was calculated based on an assumed 80% worm reduction and a variance of 30% in the group of untreated animals, thus a minimum of 6 animals were needed per group (treated vs. untreated, alpha = 0.05, beta = 0.2).

### Pilot clinical study (step 4)

An ongoing observational study conducted in Lambaréné, Gabon, assessed the effect of the treatment of acute uncomplicated malaria with antimalarial combination therapy on concomitant urogenital schistosomiasis. Here we present data from pediatric patients treated with pyronaridine-artesunate. Study participants received once-daily fixed dose pyronaridine-artesunate therapy (180 mg pyronaridine tetraphosphate plus 60 mg artesunate, Pyramax, Shin Poong Pharmaceutical Co. Ltd) for three days following label information (1 tablet for 20 to 23.9 kg, 2 tablets for 24 to 44.9 kg). Participants positive for *S*. *haematobium* eggs by urine filtration and microscopy before treatment start were included into the analysis. Urine was resampled 28 days later, and egg excretion was assessed.

### Data analysis

The response of *in vitro* schistosomula assays was either expressed as % viability (microscopy data), or as normalized relative light units (RFU) for resazurin and lactate assays. Data were analyzed by GraphPad Prism 6 v6.07 to determine the 50% inhibitory concentrations (IC50) applying a four-parameter logistic regression analysis of log-transformed drug concentration response curves. After data collection, outliers were identified by ROUT and removed (Juvenile set: 2 data points, Adult set: 1 data point). Cleaned data were analyzed and median (IQR) values were calculated. Kruskal-Wallis multiple comparison was done to identify significant differences in worm counts between compound and control group (no drug). Dunn´s test was done to correct for multiple comparisons. Adjusted P-values are reported.

## Supporting information

S1 Fig Assay quality controls of *in vitro* drug assays with schistosomula (step 1). Individual results, median and IQR of schistosomula viability in medium only and in 0.8% DMSO after 7 days *in vitro* culture (negative controls), and IC50 of mefloquine (MQ, positive control), respectively. Every drug assay included these 3 controls.
(TIF)

S2 Fig Agreement of microscopy and resazurin assay. Bland-Altman plot of difference of microscopy and resazurin assay based IC50 values obtained from schistosomula after 7 days of drug exposure. Analysis was done using individual in vitro assay outcomes of all compounds tested. Upper and lower dotted lines represent the 95% limits of agreement.
(TIF)

S3 Fig *In vitro* drug assay of *ex vivo* adult *S*. *mansoni* worms. Worms were exposed to A) no drug (negative control), B) 1 μM praziquantel, C) 30 μM pyronaridine, and D) 30 μM methylene blue, respectively, for 7 days followed by an additional 7 days without drug (drug wash-out, but medium only) to confirm the detrimental drug effect. Then viability was assessed (see photos A, B, C, and D and S1 Videos).
(TIF)

S4 Fig Liver granulation after drug treatment of *S*. *mansoni*-infected mice. Mice were infected with 100 cercariae of *S*. *mansoni*. 14 days later (when the parasite is still in at a juvenile stage), mice were exposed to A: no drug (negative control), B: artesunate, C: pyronaridine, and D: methylene blue. Mice were euthanized (CO_2_-inhalation) eight weeks post treatment and liver were taken out later to document the burden of eggs (granulation).
(TIF)

S5 Fig Dose-response curves of *in vitro* schistosomula assays (step 1) resulting from different drugs and assay read-outs. Dose-response curves of viability assessment obtained by microscopy at 72 h and at day 7, resazurin assay at day 7, and lactate assay at day 7 are shown for mefloquine, pyronaridine, and tigecycline. Every drug was measured independently three times (red, yellow, and green lines). The 50% inhibitory concentration (IC50) per drug per assay is calculated from curves following a sigmoidal dose-response, see mefloquine all assays. Pyronaridine effectively inhibited schistosomes when evaluated by microscopy. Despite a dose-dependent inhibition of the worms as shown by resazurin and lactate assays (experiments D2 and D4), the response was not sigmoidal and thus an IC50 could not be derived. Tigecycline is an example a drug not active against schistosomula.
(TIF)

S1 Table Test compounds. Drug concentration range (in μM) and dilution factor (DF) are indicated for *in vitro* drug testing against schistosomula (step 1). Each series of drug concentration tested included always a well without any drug (medium only). PC: positive control drug, Abb.: abbreviation, i.d.: in development.
(PDF)

S2 Table Published data on antimalarials that report drug activity against *S*. *mansoni*. All data are expressed in molarity and were converted (*) if necessary. WBR Worm burden reduction, LD: lethal dose, d: days, n.d.: no data was found in the literature.
(PDF)

S3 Table Baseline characteristics of study participants. The children had the following egg counts: * 4, 6, or 14 eggs/10 ml urine. ^#^: 256, 322, or 2032 eggs/10 ml urine. Infection intensity: light ≤ 50 eggs/10 ml urine, heavy > 50 eggs/10 ml urine. By Combur10 test: microhematuria: >5 ery/μl, proteinuria: > 0.3 g/l, leukocyturia: > 10 leu/μl.
(PDF)

S4 Table Activity against adult *S*. *mansoni* in mice (low infection rates). Mice infected with *S*. *mansoni* for 9 weeks (worms are in the adult stage) were treated with methylene blue (MB) or pyronaridine (PY). Negative control treatment: No drug and solvent, positive control treatment: Praziquantel (PZQ). WBR is referred to the “No drug” group. WBR: Worm burden reduction, LGS: Liver granulation score. * 1 mouse died during the treatment week. ^a^ Interquartile range: IQR. Results from one experiment are shown. Of note: These are results of the repeated experiment, but as infection rates in control mice were low, they are presented here separately.
(PDF)

S1 Data Excel spreadsheet with underlaying numerical data. Data for figures are organized in tabs.
(XLSX)

S1 Videos *Ex vivo* adult *S*. *mansoni* worms after drug exposure. Worms were exposed to S3A) no drug (negative control), S3B) 1 μM praziquantel, S3C) 30 μM pyronaridine, and S3D) 30 μM methylene blue, respectively, for 7 days followed by an additional 7 days without drug (drug wash-out, but medium only).
(ZIP)
