# Correction: Evidence for *in vitro* and *in vivo* activity of the antimalarial pyronaridine against *Schistosoma*

**DOI:** 10.1371/journal.pntd.0010512

**Published:** 2022-06-02

**Authors:** Erik Koehne, Nina Zander, Miriam Rodi, Jana Held, Wolfgang Hoffmann, Rella Zoleko-Manego, Michael Ramharter, Ghyslain Mombo-Ngoma, Peter G. Kremsner, Andrea Kreidenweiss

There is an error in Table 2. The line shifted some data into the incorrect drug candidates. Please see the correct Table 2 here.

**Table 2:** Drug activity against *ex vivo* adult worms (step 2).

Drug	Concentration in µM	No. of worms tested	% affected	% dead
No drug	NA	12	0	0
DMSO	0.1%	10	0	0
Praziquantel	1	12	0	100
Methylene blue	5	17	35	59
	10	12	0	100
	30	12	0	100
	30*	6	0	100
Pyronaridine	5	15	0	60
	10	11	45	55
	30	12	0	100
	30*	6	0	100

Methylene blue, pyronaridine, and praziquantel (positive control) were exposed to the respective concentrations for 7 days (* for 24 h) followed by a 7 days drug wash-out *in vitro* culture.”No drug” and DMSO were the negative controls. Pooled data obtained from 3 experiments per drug are displayed.
