# Supplementary figures and images for: Evidence for in vitro and in vivo activity of the antimalarial pyronaridine against Schistosoma
Source: PLoS Negl Trop Dis. 2021 Jun 24;15(6):e0009511. doi: 10.1371/journal.pntd.0009511 (PMC8263063; doi:10.1371/journal.pntd.0009511)

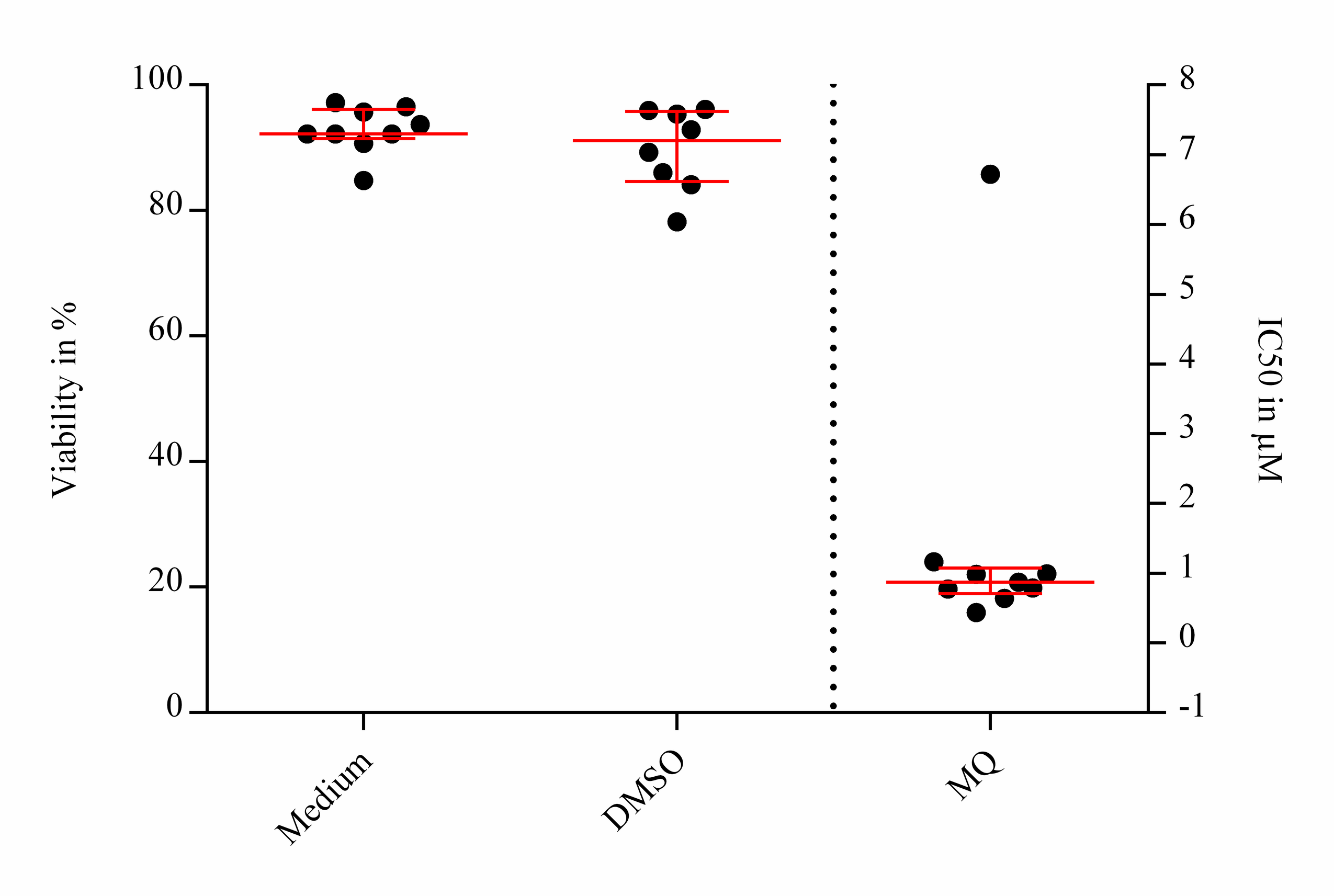

Supplement: S1 Fig — Individual results, median and IQR of schistosomula viability in medium only and in 0.8% DMSO after 7 days in vitro culture (negative controls), and IC50 of mefloquine (MQ, positive control), respectively. Every drug assay included these 3 controls. (TIF) [file pntd.0009511.s001.tif]

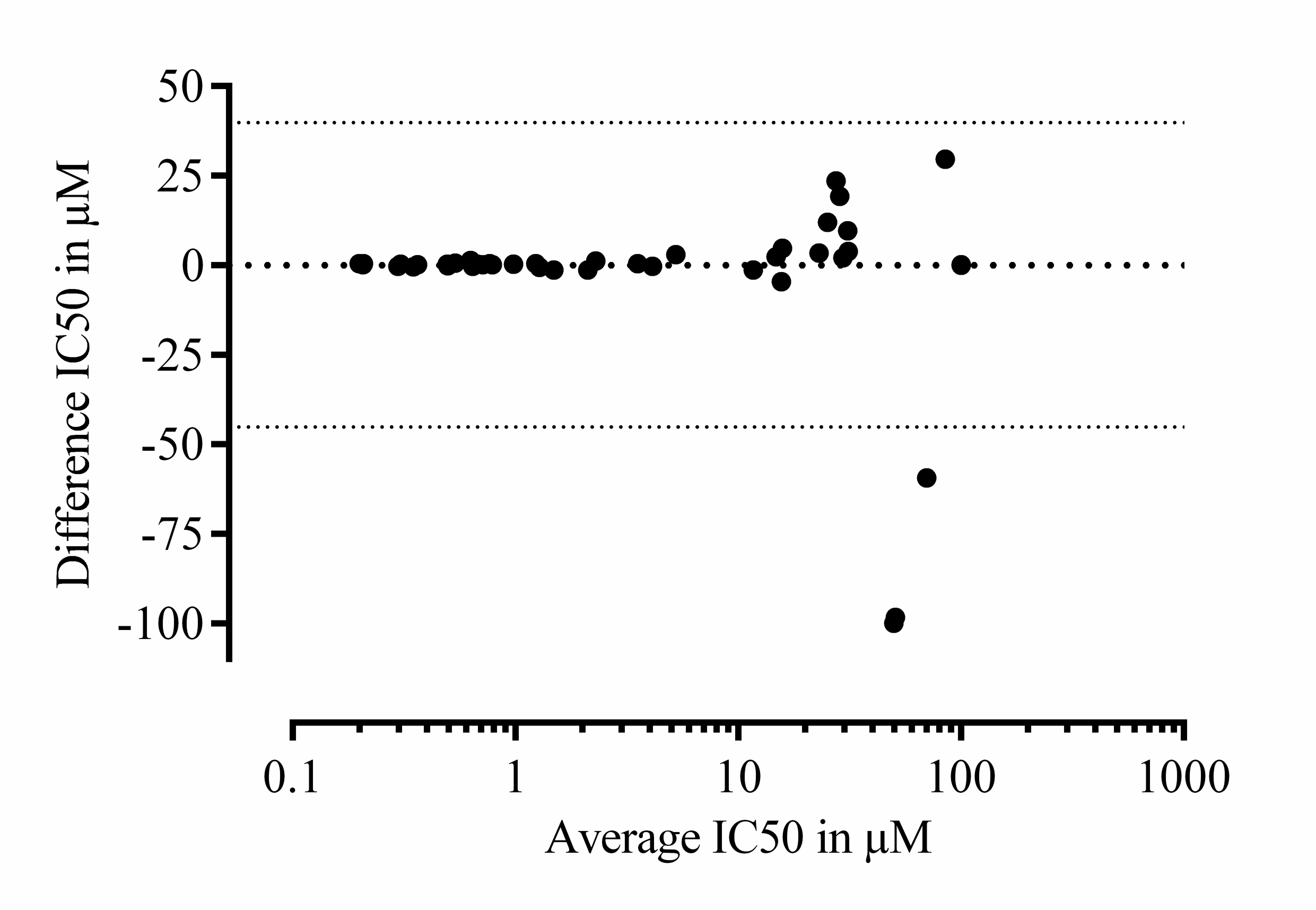

Supplement: S2 Fig — Bland-Altman plot of difference of microscopy and resazurin assay based IC50 values obtained from schistosomula after 7 days of drug exposure. Analysis was done using individual in vitro assay outcomes of all compounds tested. Upper and lower dotted lines represent the 95% limits of agreement. (TIF) [file pntd.0009511.s002.tif]

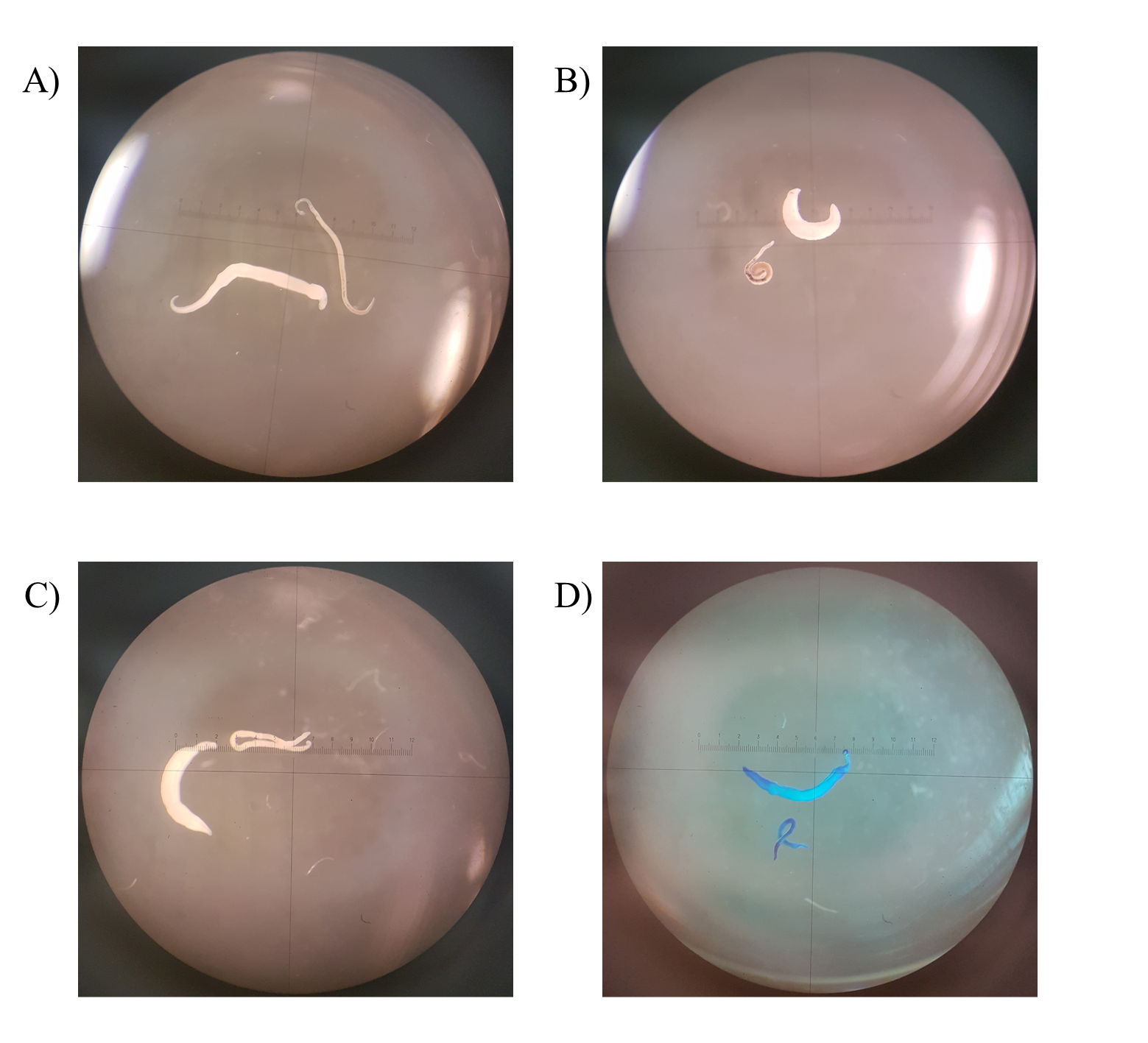

Supplement: S3 Fig — Worms were exposed to A) no drug (negative control), B) 1 μM praziquantel, C) 30 μM pyronaridine, and D) 30 μM methylene blue, respectively, for 7 days followed by an additional 7 days without drug (drug wash-out, but medium only) to confirm the detrimental drug effect. Then viability was assessed (see photos A, B, C, and D and S1 Videos). (TIF) [file pntd.0009511.s003.tif]

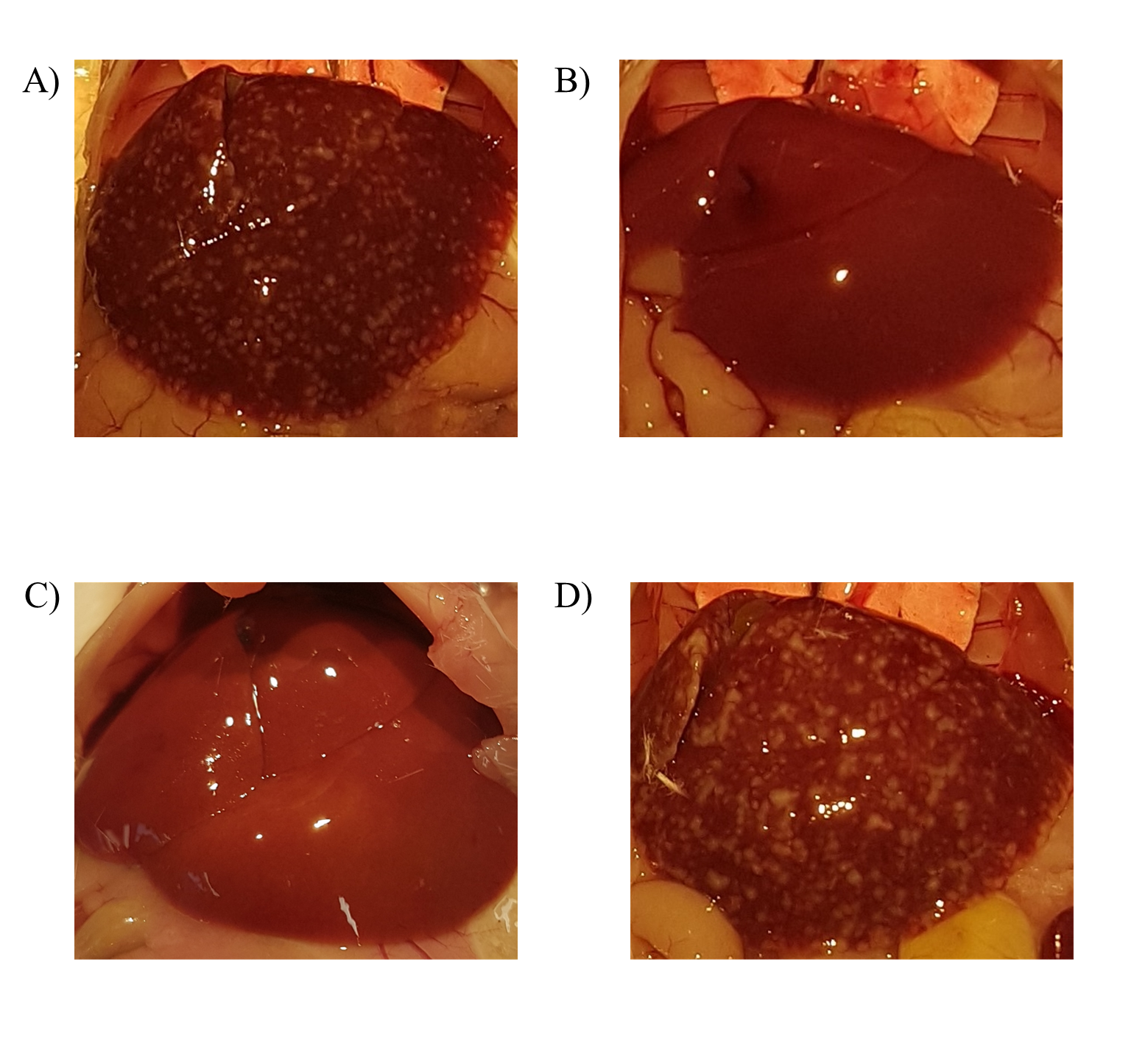

Supplement: S4 Fig — Mice were infected with 100 cercariae of S. mansoni. 14 days later (when the parasite is still in at a juvenile stage), mice were exposed to A: no drug (negative control), B: artesunate, C: pyronaridine, and D: methylene blue. Mice were euthanized (CO2-inhalation) eight weeks post treatment and liver were taken out later to document the burden of eggs (granulation). (TIF) [file pntd.0009511.s004.tif]

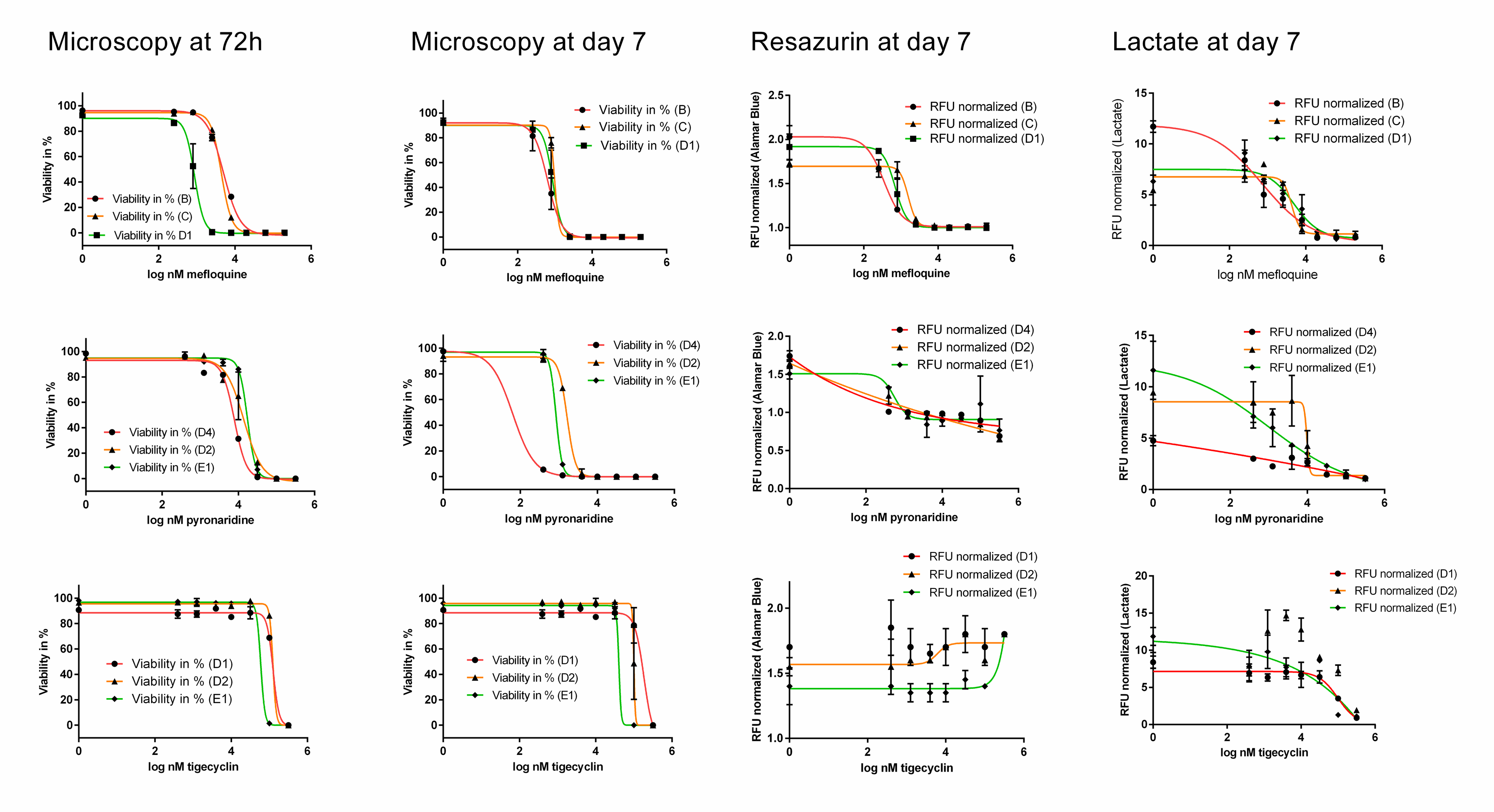

Supplement: S5 Fig — Dose-response curves of viability assessment obtained by microscopy at 72 h and at day 7, resazurin assay at day 7, and lactate assay at day 7 are shown for mefloquine, pyronaridine, and tigecycline. Every drug was measured independently three times (red, yellow, and green lines). The 50% inhibitory concentration (IC50) per drug per assay is calculated from curves following a sigmoidal dose-response, see mefloquine all assays. Pyronaridine effectively inhibited schistosomes when evaluated by microscopy. Despite a dose-dependent inhibition of the worms as shown by resazurin and lactate assays (experiments D2 and D4), the response was not sigmoidal and thus an IC50 could not be derived. Tigecycline is an example a drug not active against schistosomula. (TIF) [file pntd.0009511.s005.tif]
