# Supplementary material for: Evidence for in vitro and in vivo activity of the antimalarial pyronaridine against Schistosoma
Source: PLoS Negl Trop Dis. 2021 Jun 24;15(6):e0009511. doi: 10.1371/journal.pntd.0009511 (PMC8263063; doi:10.1371/journal.pntd.0009511)
Supplement: S1 Table — Drug concentration range (in μM) and dilution factor (DF) are indicated for in vitro drug testing against schistosomula (step 1). Each series of drug concentration tested included always a well without any drug (medium only). PC: positive control drug, Abb.: abbreviation, i.d.: in development. (PDF) [file pntd.0009511.s006.pdf]

No.	Compound	Abb.	Drug class	Concentration range tested		Approved antimalarial
				µM	DF	
PC	Mefloquine	MQ	4-quinoline-methanol	200.0 - 0.3	1:3	yes
1	Amodiaquine	AQ	4-aminoquinoline	200.0 - 3.1	1:2	yes
2	Artesunate	AS	endoperoxide	200.0 - 3.1	1:2	yes
3	Atovaquone	AV	napthoquinone	200.0 - 3.1	1:2	yes
4	Chloroquine	CQ	4-aminoquinoline	200.0 - 3.1	1:2	yes
5	Ferroquine	FQ	4-aminoquinoline	50.0 - 0.8	1:2	i.d.
6	Cycloguanil	CG	biguanide	300.0 - 0.4	1:3	no
7	Proguanil	PG	biguanide	300.0 - 0.4	1:3	yes
8	Methylene blue	MB	thiazine dye	300.0 - 0.4	1:3	i.d.
9	Primaquine	PQ	8-aminoquinoline	50.0 - 0.8	1:2	yes
10	Pyronaridine	PY	derivative of mepacrine	300.0 - 0.4	1:3	yes
11	Quinine	QN	quinoline	200.0 - 3.1	1:2	yes
12	Clindamycin	CL	antibiotic	300.0 - 0.4	1:3	yes
13	Doxycycline	DX	antibiotic	300.0 - 0.4	1:3	yes
14	4-cis-Mirincamycin	CM	antibiotic	300.0 - 0.4	1:3	no
15	4-trans-Mirincamycin	TM	antibiotic	300.0 - 0.4	1:3	no
16	Tigecyclin	TI	antibiotic	300.0 - 0.4	1:3	no
