# Supplementary material for: Evidence for in vitro and in vivo activity of the antimalarial pyronaridine against Schistosoma
Source: PLoS Negl Trop Dis. 2021 Jun 24;15(6):e0009511. doi: 10.1371/journal.pntd.0009511 (PMC8263063; doi:10.1371/journal.pntd.0009511)
Supplement: S2 Table — All data are expressed in molarity and were converted (*) if necessary. WBR Worm burden reduction, LD: lethal dose, d: days, n.d.: no data was found in the literature. (PDF) [file pntd.0009511.s007.pdf]

	In vitro		*In vitro*		*In vivo* (Schistosoma-infected mice)					
	Schistosomula		*Ex vivo* adult worms		Juvenile stages			Adult stages		
	Activity	Ref.	Activity	Ref.	Cure	WBR	Ref.	Cure	WBR	Ref.
**Mefloquine**	IC50: 6 µM (after 72 h)	[28]	IC50:11 µM (after 72 h)	[28]	1x 400 mg/kg: 20%	1x 400 mg/kg: 98%	[13]	1x 400 mg/kg: 0%	1x 400 mg/kg: 77%	[13]
**Amodiaquine**	n.d.	-	LD: 11 µM* (after 14 days)	[45]	n.d.	n.d.	-	1x 400 mg/kg: 0%	1x 400 mg/kg: 0%	[13]
**Artesunate**	IC50: 1.5 µM (after 72 h)	[46]	LD: 52 µM* (after 6 days)	[47]	-	3x 300 mg/kg: 77 %	[31]	-	2x 400 mg/kg: 33 %	[26]
**Atovaquone**	n.d.	-	n.d.	-	n.d.	n.d.	-	1x 400 mg/kg: 0%	1x 400 mg/kg: 0%	[13]
**Chloroquine**	52 µM*: no effect after 3 days	[48]	52 µM*: no effect after 3 days	[48]	n.d.	n.d.	-	1x 400 mg/kg: 0%	1x 400 mg/kg: 12%	[13]
**Ferroquine**	76 µM*: partial effect after 3 days	[48]	76 µM*: partial effect after 3 days	[48]	n.d.	n.d.	-	1x 800 mg/kg: 0%	1x 800 mg/kg: 36%	[48]
**Cycloguanil**	n.d.	-	n.d.	-	n.d.	n.d.	-	n.d.	n.d.	-
**Proguanil**	n.d.	-	n.d.	-	n.d.	n.d.	-	n.d.	n.d.	-
**Methylene blue**	10 µM: active at 72h	[19]	33 µM after 24 h: not active	[46]	n.d.	n.d.	-	n.d.	n.d.	-
**Primaquine**	4 µM: 100% dead after 3 days	[29]	4 µM: 9% dead after 7 days	[29]	n.d.	n.d.	-	n.d.	n.d.	-
**Pyronaridine**	IC50: 41 µM* 10 µM: active at 72h	[19]	33 µM after 24 h: not active	[46]	n.d.	n.d.	-	1x 400 mg/kg: 0%	1x 400 mg/kg: 0%	[13]
**Quinine**	IC50: 252 µM* after 9 days	[49]	54 µM* combined with hemin: 100% dead	[49]	n.d.	n.d.	-	1x 400 mg/kg: 0%	1x 400 mg/kg: 55%	[13]
**Clindamycin**	n.d.	-	n.d.	-	n.d.	n.d.	-	n.d.	n.d.	-
**Doxycycline**	n.d.	-	IC50: 218 µM* after 9 days	[50]	n.d.	n.d.	-	n.d.	n.d.	[50]
**cis-Mirincamycin**	n.d.	-	n.d.	-	n.d.	n.d.	-	n.d.	n.d.	-
**trans-Mirincamycin**	n.d.	-	n.d.	-	n.d.	n.d.	-	n.d.	n.d.	-
**Tigecycline**	n.d.	-	n.d.	-	n.d.	n.d.	-	n.d.	n.d.	-
