# Supplementary material for: Evidence for in vitro and in vivo activity of the antimalarial pyronaridine against Schistosoma
Source: PLoS Negl Trop Dis. 2021 Jun 24;15(6):e0009511. doi: 10.1371/journal.pntd.0009511 (PMC8263063; doi:10.1371/journal.pntd.0009511)
Supplement: S3 Table — The children had the following egg counts: * 4, 6, or 14 eggs/10 ml urine. #: 256, 322, or 2032 eggs/10 ml urine. Infection intensity: light ≤ 50 eggs/10 ml urine, heavy > 50 eggs/10 ml urine. By Combur10 test: microhematuria: >5 ery/μl, proteinuria: > 0.3 g/l, leukocyturia: > 10 leu/μl. (PDF) [file pntd.0009511.s008.pdf]

**N**	**6**	
Male/Female	5/1	
Age in years, median (range)	7.5	(4 -10)
Eggs/10 ml, median (range)	135	(4-2032)
Patients with light/heavy infection, n (%)	3*/3#	(50/50)
Patients with microhematuria, n	5	
Patients with proteinuria, n	6	
Patients with leukocyturia, n	2	
