# Supplementary material for: Evidence for in vitro and in vivo activity of the antimalarial pyronaridine against Schistosoma
Source: PLoS Negl Trop Dis. 2021 Jun 24;15(6):e0009511. doi: 10.1371/journal.pntd.0009511 (PMC8263063; doi:10.1371/journal.pntd.0009511)
Supplement: S4 Table — Mice infected with S. mansoni for 9 weeks (worms are in the adult stage) were treated with methylene blue (MB) or pyronaridine (PY). Negative control treatment: No drug and solvent, positive control treatment: Praziquantel (PZQ). WBR is referred to the “No drug” group. WBR: Worm burden reduction, LGS: Liver granulation score. * 1 mouse died during the treatment week. a Interquartile range: IQR. Results from one experiment are shown. Of note: These are results of the repeated experiment, but as infection rates in control mice were low, they are presented here separately. (PDF) [file pntd.0009511.s009.pdf]

Adult worms (9 weeks old)								
**Drug**	**Dose in mg/kg**	**No. of mice**	**No. of worms/mouse recovered Median (IQR[a])**			**Cure in (%)**	**WBR in %**	**LGS Median (IQR[a])**
			Total	Males	Females			
No drug	NA	6	3.0 (2.5-3.3)	3.0 (2.5-3.3)	0.0 (0.0-0.0)	NA	0	0.0 (0.0-0.0)
Solvent	NA	6	3.0 (1.8-4.5)	2.5 (1.8-4.5)	0.0 (0.0-0.3)	NA	0	0.0 (0.0-0.0)
Praziquantel	500	6	0.0 (0.0)	0.0 (0.0)	0.0 (0.0)	100	100	0.0 (0.0-0.0)
Pyronaridine	500	6*	3.0 (2.5-4.5)	3.0 (2.5-4.5)	0.0 (0.0-0.0)	0	0	0.0 (0.0-0.0)
Methylene blue	50	6	1.0 (0.0-2.3)	1.0 (0.0-2.3)	0.0 (0.0-0.0)	33	59	0.0 (0.0-0.0)
